# CUDC-907, a dual PI3K/histone deacetylase inhibitor, increases *meta*-iodobenzylguanidine uptake (^123/131^I-mIBG) in vitro and in vivo: a promising candidate for advancing theranostics in neuroendocrine tumors

**DOI:** 10.1186/s12967-023-04466-z

**Published:** 2023-09-07

**Authors:** Joana Grand-Guillaume, Rosalba Mansi, Raghuvir H. Gaonkar, Sandra Zanger, Melpomeni Fani, Philippe J. Eugster, Maja Beck Popovic, Eric Grouzmann, Karim Abid

**Affiliations:** 1 Catecholamine and Peptides Laboratory, Service of Clinical Pharmacology and Toxicology, Lausanne University Hospital and University of Lausanne, 1011 Lausanne, Switzerland; 2 Division of Radiopharmaceutical Chemistry, Clinic of Radiology and Nuclear Medicine, University Hospital Basel, 4031 Basel, Switzerland; 3 Pediatric Hematology-Oncology Unit, Woman-Mother-Child Department, Lausanne University Hospital and University of Lausanne, Lausanne, Switzerland

**Keywords:** Neuroendocrine tumors, Theranostics, *Meta*-iodobenzylguanidine (mIBG), Histone deacetylase inhibitor (HDACi), PI3K/AKT/mTOR inhibitor, Xenograft

## Abstract

**Background:**

Neuroblastoma (NB) and pheochromocytoma/paraganglioma (PHEO/PGL) are neuroendocrine tumors. Imaging of these neoplasms is performed by scintigraphy after injection of radiolabeled *meta*-iodobenzylguanidine (mIBG), a norepinephrine analog taken up by tumoral cells through monoamine transporters. The pharmacological induction of these transporters is a promising approach to improve the imaging and therapy (theranostics) of these tumors.

**Methods:**

Transporters involved in mIBG internalization were identified by using transfected Human Embryonic Kidney (HEK) cells. Histone deacetylase inhibitors (HDACi) and inhibitors of the PI3K/AKT/mTOR pathway were tested in cell lines to study their effect on mIBG internalization. Studies in xenografted mice were performed to assess the effect of the most promising HDACi on ^123^I-mIBG uptake.

**Results:**

Transfected HEK cells demonstrated that the norepinephrine and dopamine transporter (NET and DAT) avidly internalizes mIBG. Sodium-4-phenylbutyrate (an HDACi), CUDC-907 (a dual HDACi and PI3K inhibitor), BGT226 (a PI3K inhibitor) and VS-5584 and rapamycin (two inhibitors of mTOR) increased mIBG internalization in a neuroblastoma cell line (IGR-NB8) by 2.9-, 2.1-, 2.5-, 1.5- and 1.3-fold, respectively, compared with untreated cells. CUDC-907 also increased mIBG internalization in two other NB cell lines and in one PHEO cell line. We demonstrated that mIBG internalization occurs primarily through the NET. In xenografted mice with IGR-NB8 cells, oral treatment with 5 mg/kg of CUDC-907 increased the tumor uptake of ^123^I-mIBG by 2.3- and 1.9-fold at 4 and 24 h post-injection, respectively, compared to the untreated group.

**Conclusions:**

Upregulation of the NET by CUDC-907 lead to a better internalization of mIBG in vitro and in vivo.

**Supplementary Information:**

The online version contains supplementary material available at 10.1186/s12967-023-04466-z.

## Background

Neuroendocrine tumors are neoplasms originating from cells of the endocrine and nervous systems that develop in organs, such as the gastrointestinal tract, pancreas, adrenal glands, lungs, and others [1]. Among them, neuroblastoma (NB) represents 12% of all pediatric cancer fatalities and may develop in various sites, although the most common site is the adrenal gland, which accounts for approximately 46% of cases [2–4]. NB may have an aggressive nature with a high propensity to metastasize. Pheochromocytomas (PHEO) are located within the adrenal medulla, while paragangliomas (PGL) are found in the sympathetic and parasympathetic ganglia [5]. PHEO/PGL are benign in nature, although approximately 10% of the cases also develop a malignancy [6, 7]. One of the biggest challenges in NB and PHEO/PGL is to assess the presence of metastasis and to find effective therapies as current treatments for malignant forms have been shown to be insufficient in terms of outcomes [2, 3].

NB and PHEO/PGL cells are characterized by an excessive production and secretion of catecholamines. Upon release, a portion of these catecholamines is taken up by cells through monoamine membrane transporters present on chromaffin and tumor cells.

Certain treatments of NB and PHEO/PGL take advantage of the monoamine transporters for a localized and specific intervention, such as the endoradiotherapy using *meta*-iodobenzylguanidine (mIBG) [8, 9]. mIBG is labeled with either ^123^I (gamma radiation) or ^131^I (beta radiation) when used for nuclear imaging and endoradiotherapy respectively [9, 10]. mIBG is internalized by norepinephrine transporters (NET), however, discrepancies between NET expression and mIBG internalization have been reported [10–13]. Therefore, we hypothesized that other monoamine transporters, such as the dopamine transporters (DAT), the plasma membrane monoamine transporters (PMAT), or the organic cation transporters (OCT1–3), may also be involved in mIBG internalization as their affinity for catecholamines has been demonstrated in several studies [14–16].

Expressions of monoamine transporters like NET and/or DAT can be pharmacologically induced by several histone deacetylase inhibitors (HDACi), such as vorinostat, sodium-4-phenylbutyrate (sodium-4-P), valproate acid, and trichostatin A [17–19]. Yet, these studies have been performed with a very limited number of HDACi and have not explored in details the identity of the transporters involved in mIBG uptake. The aim of the present study was first to identify possible additional monoamine transporters involved in mIBG internalization and second to pharmacologically upregulate the synthesis of these transporters to increase mIBG internalization into a translational approach from cell lines to a mice model.

## Methods

### Drugs and reagents

mIBG was purchased from Axon Medchem, deuterated mIBG from Alsachim and all the inhibitors were from Selleckchem, Medchem-Express or Sigma-Aldrich (Additional file 1: Table S2). Hygromycin B, penicillin/streptomycin mixture, Tween-20, bovine serum albumin (BSA) and paraformaldehyde were purchased from Sigma-Aldrich. Dulbecco’s Modified Eagle Medium (DMEM)-GlutaMAX and geneticin were purchased from Life Technology and fetal bovine serum (FBS) from Biowest. Methanol, formic acid and acetonitrile were purchased from Biosolve and ammonium formate from Fluka. N-glycosidase F and protease inhibitors were purchased from Roche and Triton X-100 from PanReac Applichem. Mouse monoclonal anti-NET antibody was purchased from MAb Technologies (#NET17-1) and rabbit polyclonal anti-DAT (ab1766) and monoclonal anti-β-actin antibody (#AC-15) from Sigma-Aldrich. Secondary HRP conjugated anti-mouse and anti-rabbit antibodies were purchased from Bio-Rad (reference 170-6516 and 170-6515, respectively).

### Human tumor specimens

Fresh tumor specimens were obtained following the surgery of 23 patients with histologically confirmed PHEO/PGL. Tumor tissues were processed by a pathologist. The 12 women and 11 men had a mean age of 56 ± 13.8 years (range 29 to 78 years). All patients were chosen to represent sporadic cases without known mutations (Additional file 1: Table S1). Two PHEOs were extra-adrenal (PGLs), while four of them were metastatic. Malignancy was proven by radiological evidence of multiple pulmonary and hepatic lesions and lymph node metastases. This study was approved by the local ethics committee for the Canton de Vaud (Reference number: 95/04) and all patients gave their consent to participate in the study.

### Cell lines and cell cultures

Human Embryonic Kidney 293 (HEK293) cells stably transfected with plasmids encoding the human DAT were kindly offered by Prof. M. Reith of the University of New York, and HEK293 cells expressing OCT1–3 and PMAT were kindly provided by Prof. J. Wang of the University of Washington. The plasmid encoding for NET was a kind gift from Prof. R. Blakely of Florida Atlantic University. SK-N-Be2C, LAN-1 (two human neuroblastoma cell lines) and PC-12 (a rat pheochromocytoma cell line) were kindly offered by Dr. Annick Mühlethaler-Mottet of the Centre Hospitalier Universitaire Vaudois and IGR-NB8 cells were from Gustave Roussy Institute (Villejuif, France). Cells were cultivated in DMEM GlutaMAX supplemented with 10% FBS, 1% of penicillin/streptomycin and 500 μg/mL geneticin or 150 μg/mL hygromycin B (for HEK-transfected cells).

### Immunoblotting

Tumor tissues were lysed in a buffer containing 0.1% SDS in PBS and then sonicated using a Branson 450 Digital Sonifier. Samples were clarified by a centrifugation step of 2000×*g* for 30 s, and the pellet was discarded. To detect NET and DAT expressions, proteins were deglycosylated. Firstly, proteins were denatured with β-mercaptoethanol 5 min at 95 °C and secondly mixed with 5% NP40, 0.4 M NaPO_4_ pH 7.4, protease inhibitors and N-glycosidase F and incubated 4 h at 37 °C. Samples were heated for 2 min at 100 °C and 10 min at 65 °C for NET and DAT detection, respectively. The Immunoblotting protocol is described elsewhere [20].

### RNA extraction and RT-qPCR

RNA from IGR-NB8 cells was extracted as previously described [20]. cDNA synthesis was performed with the PrimeScript Reverse Transcriptase Kit (Takara Bio Inc). PCR was performed by using the SYBR Green Master Mix (Roche) for NET, DAT, PMAT, OCT1–3, glyceraldehyde 3-phosphate dehydrogenase (GAPDH) and eukaryotic translation elongation factor 1 alpha 1 (EEIF1A1). Expression levels of NET and DAT transcripts were calculated relative to the level of the housekeeping genes GAPDH and EEIF1A1 using the ΔΔCt method [21].

### mIBG internalization in cell lines

In all experiments, cells were seeded in 48-well plates and grown at 37 °C for 24 h. mIBG internalization was blocked by rapid removal of the medium containing mIBG and by adding cold PBS for three washes before lysing the cells with 100 µL 0.2% Tween-20 in PBS. Intracellular and extracellular mIBG were then extracted and quantified. Concentrations were normalized to the total protein content in each sample using the bicinchoninic acid (BCA) protein assay (ThermoScientific).

#### Time course

mIBG was added for 0, 1 and 4 h at a concentration of 100 nM.

#### Kinetics

HEK-transfected cells were incubated with mIBG at different concentrations (from 0 to 20 µM) for 10 min at 37 °C.

#### Inhibitors

Specificity of DMI and GBR12935 toward NET and DAT, respectively, was determined by increasing concentrations of inhibitors (from 0 to 10 µM) at 37 °C, 30 min before mIBG incubation. To study the effect of NET and DAT inhibition on mIBG internalization in CUDC-907-treated cells, cells were incubated with CUDC-907 0.1 µM for 48 h following DMI and/or GBR12935 treatment at different concentrations (from 0 to 1 µM) for 30 min prior to mIBG incubation (10 nM for 10 min). For testing HDAC and PI3K/AkT/mTOR inhibition effects, cells were treated for 48 h at 37 °C and incubated with mIBG 10 nM for 10 min.

#### Assessment of the mIBG on-rate

IGR-NB8 cells were treated with CUDC-907 for 48 h and mIBG 10 nM was added in the different wells for 10 min. Cells were then washed with pre-warmed PBS and finally culture medium with or without CUDC-907 0.1 µM was added to the cells for a further incubation time corresponding to 5 min, 1, 6, 12, 24 and 48 h.

### Proteomic

HEK293 transfected cells were lysed in a buffer containing 0.1% SDS and total protein fractions were analyzed and quantified by Liquid Chromatography coupled to tandem Mass Spectrometry (LC–MS/MS) at the laboratory of Protein Analysis Facility at the University of Lausanne. The detailed procedure is described in the Additional file 2: Material and Methods section.

### Immunofluorescence

IGR-NB8 cells were seeded on coverslips coated with poly-l-lysine (Sigma-Aldrich) in 24-well cell culture plates and treated with CUDC-907 for 48 h. NET and DAT proteins were revealed after incubation with primary antibodies (ref. NET17-1 and ab1766) and Alexa FluorTM-coupled secondary antibodies (ref. A11001 and A 11008, anti-mouse and anti-rabbit respectively) from ThermoFischer. Specificity was assessed by primary antibody omission. The immunofluorescence protocol is described elsewhere [22]. Cells were imaged using a fluorescence microscope (Leica DFC 345 FX) and then analyzed with LAS AF Lite software (Version 2.6.0, build 7266). Signal was quantified using the ImageJ software.

### siRNA

IGR-NB8 cells were plated in a 24-well cell culture plate (9 × 10^4^ cells) 24 h before the experiment. Cells were then transfected with the following 25 nM siRNA mixtures: ON-TARGETplus SMARTpool siRNA mixtures targeting NET, DAT, targeting control siRNA and GAPDH control siRNA from Dharmacon, (PerkinElmer). 24 h later, cells were treated with CUDC-907 0.1 µM or BGT226 0.05 µM for another 48 h and in experiments involving mIBG uptake, incubated with 10 nM mIBG at 37 °C for 10 min before mIBG extraction and quantification.

### mIBG extraction and LC–MS/MS quantification

mIBG was extracted from cells and media by solid-phase extraction and quantified by LC–MS/MS. The detailed procedure is described in the Additional file 2: Material and Methods section.

### Animal studies

All animal experiments were carried out with female athymic nude-*Foxn1*^*nu*^*/Foxn1*^+^mice (Envigo) that were approved by the Veterinary Office of the Cantonal Basel-Stadt (Approval no 32562) in accordance with the Swiss regulations for animal treatment. The mice (4–6 weeks old) were subcutaneously implanted with 2 × 10^6^ IGR-NB8 cells/mouse in DMEM/Matrigel (1/1 v/v, 200 µL) and monitored twice a week. The tumors reached a size of 120–200 mm^3^ in 1 month, after which the mice were used for the study.

### Treatment and biodistribution studies of ^123^I-mIBG

Animals were randomly divided into three groups: group A (n = 7) received the vehicle (10% DMSO in corn oil), group B (n = 9) received 5 mg/kg of CUDC-907 and group C (n = 13) received 10 mg/kg of CUDC-907. CUDC-907 was administered for 5 days, via oral gavage, at the indicated doses and after 2 days drug-free the mice were injected with ^123^I-mIBG (2–4 MBq/100 µL). Quantitative biodistribution studies were performed 4 h and 24 h post-injection (p.i.) of ^123^I-mIBG via the tail vein. All mice were administered intravenously with sodium perchlorate (100 μL Irenat, 120 mg/kg) 5 min before ^123^I-mIBG injection for blocking the uptake of free radioiodine in iodine-avid organs. ^123^I-mIBG (AdreViewTM) is produced by GE Healthcare (Chicago, Illinois, USA) and was purchased from Medeo (Schöftland, Switzerland). Based on the specification given for it (74 MBq/0.08 mg in 1 mL), the radioligand was diluted with 0.9% NaCl in order to obtain the desired activity for the different experiments performed, namely 2–4 MBq/2–4 µg of mIBG for biodistribution studies and 13–17 MBq/14–18 µg of mIBG for SPECT studies.

### Single-photon emission computed tomography/Computed tomography (SPECT/CT) imaging

SPECT/CT images were acquired at 4 h and 24 h p.i. of ^123^I-mIBG (13–17 MBq/100 µL) using a dedicated small animal scanner (Nano-SPECT/CT™ Bioscan Inc.). The SPECT/CT images 4 h p.i. were acquired for 90 min with the mice under anesthesia. SPECT/CT images of the same mice were also acquired at 24 h p.i. for 170 min. The mice were euthanised thereafter. Procedures are described in greater detail on Additional file 2: Material and Methods section.

### Data analysis

Data are presented as the mean ± standard deviation (SD). Statistical analyses were carried out using GraphPad Prism 9.0. Statistical analysis were performed through either two-way ANOVA or unpaired t-test. P-value of less than 0.05 was considered statistically significant.

## Results

### Assessment of the correlation between NET and DAT protein expression and ^123^I-mIBG uptake

**Fig. 1:**
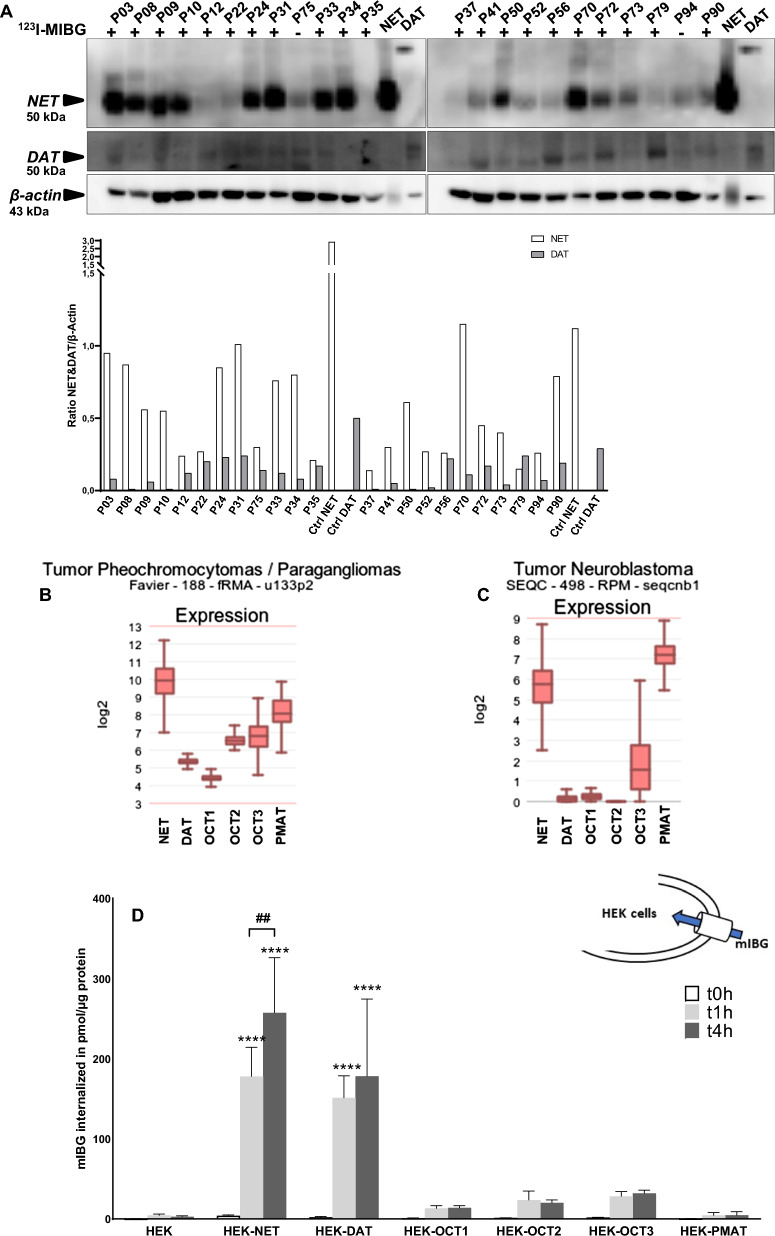

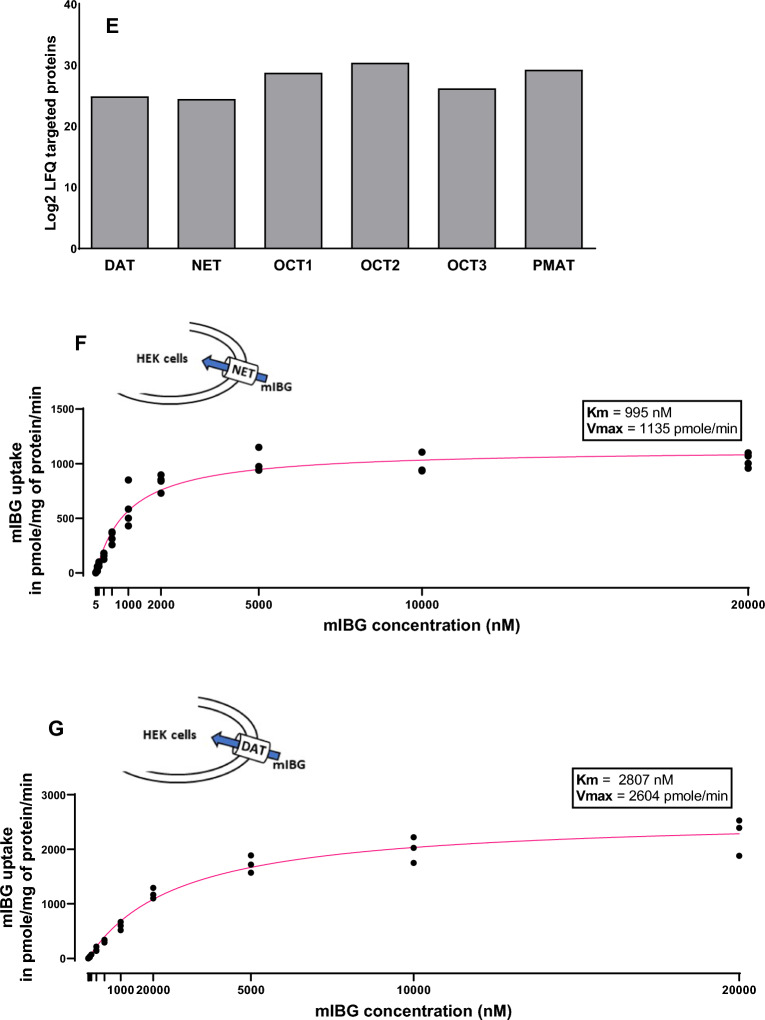
NET and DAT protein expression and ^123^I-mIBG internalization in PHEO tumor biopsies and cellular mIBG internalization in HEK cells transfected with monoamine transporters. **A** Immunoblotting analysis of NET and DAT in PHEO tumor biopsies from patients. HEK-NET and HEK-DAT protein extracts were used as positive and negative controls for NET and DAT protein expression, and β-actin was used as a loading control. Quantification of immunoreactive signal was normalized with β-actin protein expression using the Image J software. **B** and **C** Illustration of the mRNA expression levels (normalized expression, in log2) of the indicated genes in one PHEO/PGL and one NB transcriptomic datasets analyzed by RNAseq from the R2: Genomics Analysis and Vizualization Platform (http://r2.amc.nl, MegaSampler analysis: Human Genome U133, Plus 2.0; MAS5.0 data normalization). **D** mIBG internalization in HEK-transfected cells incubated with mIBG 100 nM during 0, 1, and 4 h. Results were normalized with a BCA test. The experiments were performed three times in triplicate. Statistical analysis was performed through two-way ANOVA between the control (HEK) and each cell line. ****p < 0.0001; an between time points 1 and 4 h, ^##^p < 0.01. *p* values are reported only when statistically significant (< 0.05). **E** Proteomic analysis of total protein fractions of HEK-transfected cells by LC–MS/MS. LFQ, label-free quantification. **F** and **G** Determination of NET and DAT Km and Vmax toward mIBG. HEK-NET/DAT results were normalized with a BCA test and performed three times in duplicates, with the mean values of each series recorded. Non-linear regression was drawn using GraphPad Prism’s Michaelis–Menten interpolation

PHEO tumor biopsies from patients having ^123^I-mIBG-positive or ^123^I-mIBG-negative (lack of) uptake were analyzed by immunoblotting to determine the expression levels of NET and DAT (Fig. 1A). Twenty-one ^123^I-mIBG-positive and two ^123^I-mIBG-negative (P75 and P94) samples were analyzed. NET protein expression was highly variable among the different samples. ^123^I-mIBG tumor uptake, detected by SPECT did not necessarily correlate with NET expression. P12 and P22 samples among others have low or non-detected NET expression but were ^123^I-mIBG-positive. Meanwhile, the two samples of ^123^I-mIBG-negative (P75 and P94) had moderate expression of NET. No correlation was observed between DAT expression and ^123^I-mIBG tumor uptake in the biopsies tested. NET, DAT, OCT1–3 and PMAT mRNA levels were compared in two transcriptomic datasets of PHEO/PGL and NB from the R2 platform (http://r2.amc.nl), and revealed that NET was significantly more expressed than DAT in both type of tumors [23, 24]. PMAT mRNA level was also significantly higher than OCT1-3 (Fig. 1B and C). Overall, this data suggests that other monoamine transporters, apart from NET and DAT, could be involved in mIBG tumor uptake such as PMAT.

### Evaluation of the contribution of each transporters for mIBG internalization in HEK-transfected cells

To investigate the involvement of different monoamine transporters in mIBG uptake, HEK cells were transfected with plasmids encoding the human forms of NET, DAT, OCT1–3 and PMAT transporters, which have been reported to display affinity for catecholamines [14–16]. mIBG internalization by NET was confirmed using HEK-NET at two time points (t1h = 178 ± 36 pmol/μg protein, fold change compared to non-transfected cells = 40 ± 8; p < 0.0001; t4h = 257 ± 69 pmol/μg protein, fold change = 92 ± 25; p < 0.0001). HEK-DAT showed a significant increase of mIBG internalization, compared to non-transfected cells, demonstrating that mIBG is also internalized by DAT (t1h = 151 ± 28 pmol/μg protein, fold change = 34 ± 6; p < 0.0001; t4h = 178 ± 96 pmol/μg protein, fold change = 63 ± 34; p < 0.0001) (Fig. 1D). HEK-OCT1-3 and HEK-PMAT were also able to internalize mIBG, but this difference was statistically insignificant compared to non-transfected cells. A proteomic approach described in the Additional file 2: material section was used to quantify transporter proteins in each cell line. Protein levels were shown to be similar, demonstrating that our results on mIBG internalization were based on transporter specificity toward mIBG, rather than the amount of transporter expressed in each cell line (Fig. 1E).

Since the involvement of OCT1–3 and PMAT on mIBG uptake was reasonably excluded, we focused on the kinetic values of NET and DAT to estimate the affinity (Km) and capacity (Vmax) toward mIBG (Fig. 1F and G, respectively). HEK-NET showed higher affinity and lower capacity (Km = 995 nM and Vmax = 1135 pmol/min) to mIBG than HEK-DAT (Km = 2807 nM and Vmax = 2604 pmol/min).

### Assessment of the specificity of NET and DAT toward mIBG internalization

**Fig. 2:**
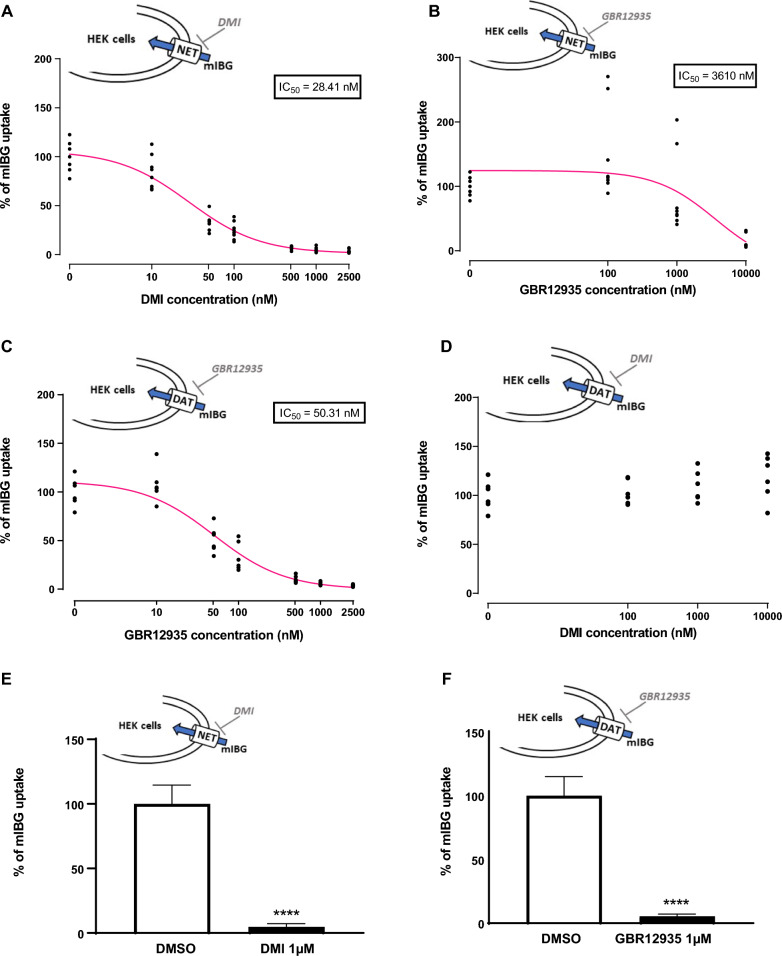
Effect of DMI and GBR12935 on mIBG internalization in HEK-NET and HEK-DAT cells. **A**–**D** mIBG internalization in HEK-NET and HEK-DAT cells treated with DMI or GBR12935. Curves were obtained through nonlinear regression and fit function of GraphPad Prism 9.1. 100% represent mIBG 10 nM, as the concentration added in the cell medium. **E** and **F** mIBG internalization in HEK-NET and HEK-DAT cells treated with or without DMI and GBR12935. Results were normalized by a BCA test. Statistical analysis was performed using the unpaired t-test between the control and condition tested. ****p < 0.0001. All the experiments were repeated at least three times in duplicates, with the mean values of each series shown for **A**–**D**

DMI and GBR12935 are NET- and DAT-specific inhibitors, respectively [25, 26]. In HEK-NET cells incubated with DMI, a very efficient inhibition of mIBG internalization was observed (IC_50_ = 28.41 nM) (Fig. 2A), while after incubation with GBR12935, inhibition on NET was very low (IC_50_ = 3610 nM) (Fig. 2B). In HEK-DAT cells incubated with GBR12935, inhibition of mIBG internalization was measured (IC_50_ = 50.31 nM) (Fig. 2C), while DMI did not affect DAT activity (Fig. 2D). Thus, the use of DMI and GBR12935 almost entirely inhibited the internalization of mIBG, in NET and DAT-expressing cells, respectively (95% for HEK-NET and 94% for HEK-DAT) when compared to the control, respectively (Fig. 2E and F). This demonstrated the specificity of NET and DAT toward mIBG.

### Evaluation of different HDACi treatments to increase mIBG internalization in distincts neuroendocrine tumor cells lines

**Fig. 3:**
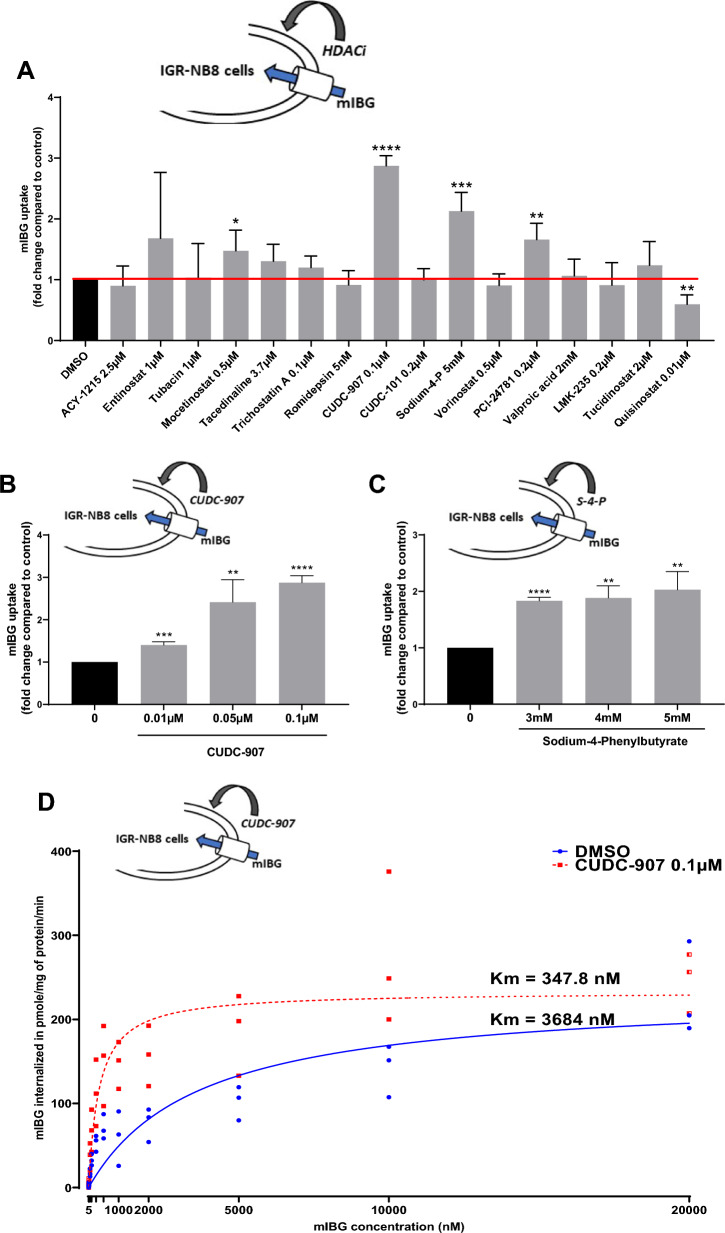

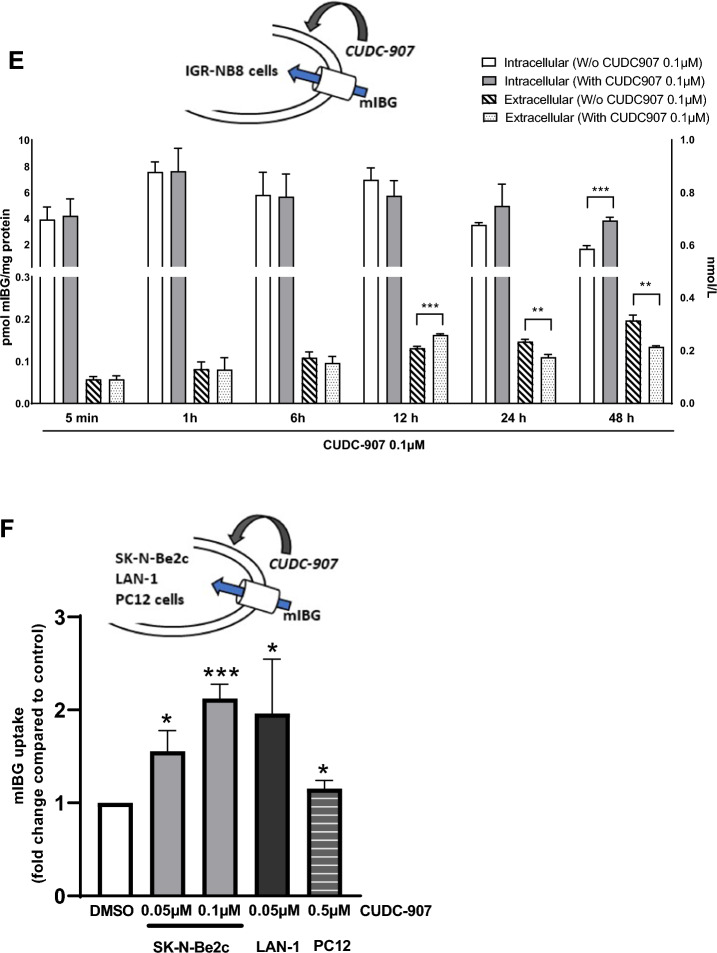
Screening of different HDACi on IGR-NB8 cells and effect of CUDC-907 on mIBG internalization. **A** mIBG internalization in IGR-NB8 cells incubated with different HDACi for 48 h. Results are expressed as fold of increase in mIBG internalization compared with non-incubated cells. **B** and **C** Dose response studies for CUDC-907 and sodium-4-P. **D** Determination of the affinity of IGR-NB8 cells toward mIBG with or without CUDC-907 treatment. Cells were incubated with DMSO or CUDC-907 0.1 µM for 48 h and then with mIBG at increasing concentrations for 10 min (X-axis). Results were normalized by a BCA test and performed three times in duplicates, with the mean values of each series shown. Non-linear regression was drawn using GraphPad Prism’s Michaelis–Menten interpolation. **E** Extracellular and intracellular mIBG concentration in IGR-NB8 cells treated with CUDC-907 over time. Y-axis on the left corresponds to intracellular mIBG concentration and is expressed as pmol of mIBG per mg of protein. Y-axis on the right corresponds to extracellular mIBG concentration and is expressed as nmol of mIBG per liter. **F** mIBG internalization in SK-N-Be2C, LAN1 and PC12 cells incubated with different concentrations of CUDC-907 for 48 h. For the experiments **A**, **B**, **C**, **E**, and **F** results were normalized by a BCA test and performed three times in duplicates. Statistical analysis was performed through the unpaired t-test between the control and condition tested. *p < 0.05; **p < 0.01; ***p < 0.001; ****p < 0.0001

Sixteen inhibitors (Fig. 3A), targeting histone deacetylase class I (HDAC1, 2, 3 and 8), IIa (HDAC4, 5, 7, 9), IIb (HDAC6 and 10) and IV (HDAC11) were tested for their effects on mIBG internalization in IGR-NB8 cells, a NB cell line, which expresses NET and DAT at the mRNA level (detected at 24.4, 29.2 and 23.3 PCR cycles for respectively NET, DAT and GAPDH). IGR-NB8 cells were incubated for 48 h with a panel of different HDACi (Fig. 3A), including vorinostat that has previously been shown to increase NET expression at the mRNA and protein levels in two different NB cell lines and on the xenografted mice model [18]. The inhibitors concentrations tested were chosen on the basis of their IC_50_ (available on the manufacturer’s website) and their use in the literature on cultured cells. Different working concentrations were tested on IGR-NB8, SK-N-Be2C, LAN-1 and PC-12 cells, and the highest tolerated concentration (based on the trypan blue assay) was selected. Mocetinostat and PCI-24781 slightly increased mIBG internalization compared to non-treated cells (fold change = 1.47 ± 0.34; p < 0.05 and 1.67 ± 0.27; p < 0.01, respectively), while two other HDACi (CUDC-907 and sodium-4-P) had a significantly stronger effect (fold change = 2.87 ± 0.17; p < 0.0001 and 2.13 ± 0.31; p < 0.001, respectively) (Fig. 3A). These two candidates were further investigated and showed a dose-dependent effect on mIBG internalization (Fig. 3B and C). All HDACi tested had no toxic effect in IGR-NB8 cells, as indicated with a trypan blue assay performed using a standard protocol [27] (Additional file 1: Fig. S1A).

Focusing on CUDC-907, we further investigated the difference of mIBG affinity for cells treated with CUDC-907 vs control cells and observed a 10.6-fold increase (348 vs 3684 nM, respectively) in the Km values (Fig. 3D).

After the initial 48 h incubation of IGR-NB8 cells with CUDC-907, assessment of the mIBG on-rate (Fig. 3E) demonstrated that intracellular mIBG concentration remains unchanged up to at least 24 h with or without continuous CUDC-907 treatment. At 48 h we observed a slight, but significant, decrease of intracellular mIBG in CUDC-907 non-treated cells compared with treated cells (1.67 ± 0.25 pmol mIBG/mg protein and 3.85 ± 0.25 pmol mIBG/mg protein, respectively, p = 0.0004). We also observed, at this time point, that extracellular mIBG in CUDC-907 treated cells remained lower compared with CUDC-907 non-treated cells (0.13 ± 0.003 pmol mIBG/mg protein, 0.20 ± 0.01 pmol mIBG/mg protein, respectively, p = 0.0011). These results demonstrate that mIBG is efficiently stored in the cytoplasm of CUDC-907 treated cells for at least 48 h after initial incubation.

The results were confirmed in two other NB cell lines, SK-N-Be2C and LAN-1, where CUDC-907 had similar effects with an increase of mIBG internalization of 2.12 ± 0.15, (p = 0.0002) and 1.96 ± 0.58, (p = 0.0167) fold, respectively, compared to non-treated cells. In addition, we also observed a slight, but significant, increase of mIBG internalization compared to control cells (fold change = 1.15 ± 0.09, p = 0.0132) in PC-12, a PHEO cell line from rat (Fig. 3F). CUDC-907 had no toxic effect in all cell lines tested (Additional file 1: Fig. S1B–D).

### Testing of the cellular effects of CUDC-907 treatment on mIBG internalization

**Fig. 4:**
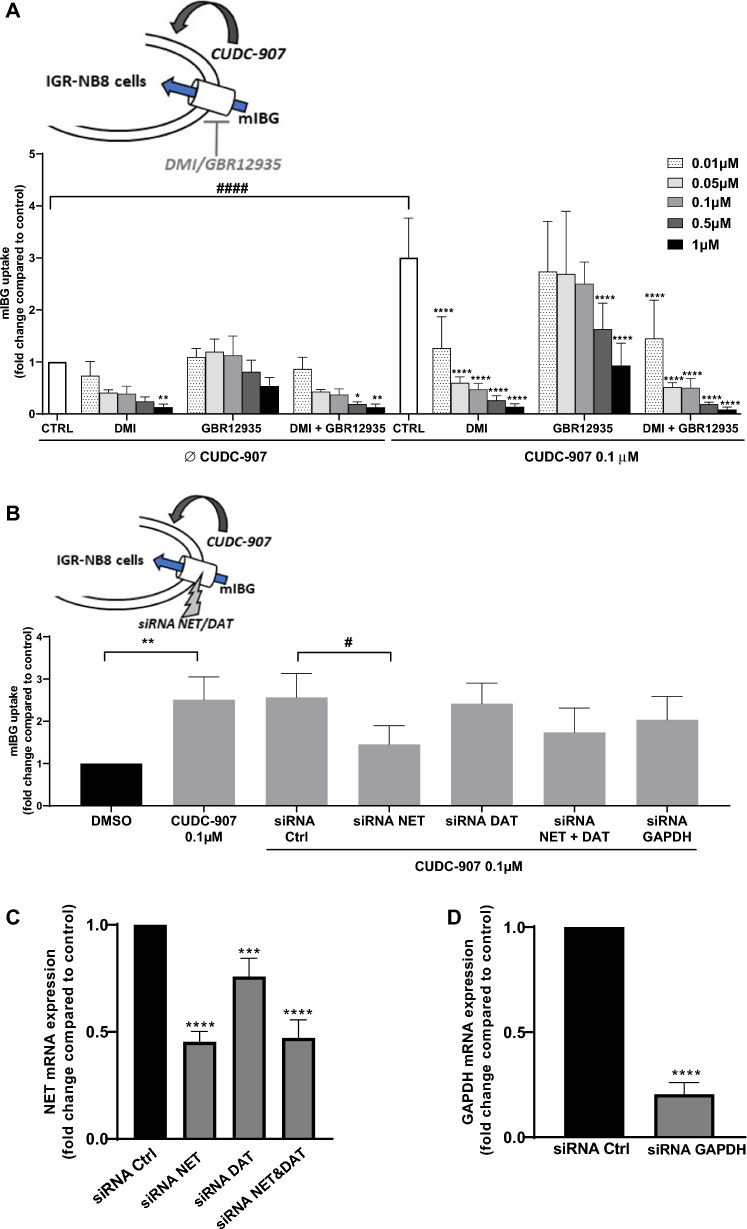

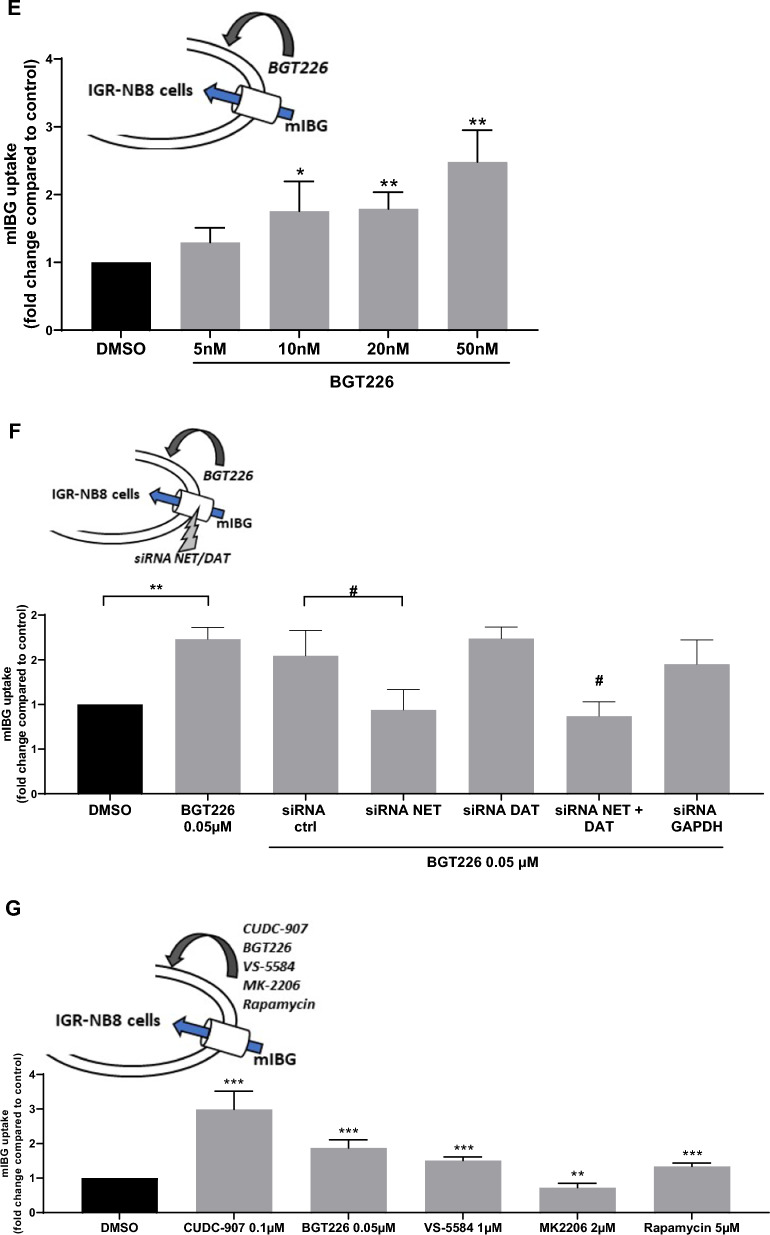
Cellular mIBG internalization in IGR-NB8 cells treated with CUDC-907 and several classes of inhibitors. **A** mIBG internalization in IGR-NB8 cells incubated with DMI, GBR12935 or a combination of both inhibitors with different concentrations (0.01, 0.05, 0.1, 0.5 and 1 μM). Statistical analysis was performed through two-way ANOVA between the respective control (with or without CUDC-907 0.1 μM) and each condition tested. *p < 0.05; **p < 0.01; ****p < 0.0001. Statistical analysis between controls with or without CUDC-907 were performed through the unpaired t-test. ^####^p < 0.0001. **B** mIBG internalization in IGR-NB8 following NET and/or DAT downregulation and CUDC-907 treatment. Statistical analysis was performed through the unpaired t-test using either DMSO (*) or siRNA non-targeting sequence (SiRNA ctrl) (#) as control and comparing with each of the conditions tested. **p < 0.01; ^#^p < 0.05. siRNA ctrl is a non-targeting sequence used as a negative control to distinguish specific and non-specific silencing. **C** and **D** qPCR quantification of NET and GAPDH mRNA concentration in IGR-NB8 transfected by siRNA. DAT mRNA signal was under the limit of quantification (> 33 PCR cycles for all conditions). Reference genes used were GAPDH and EEIF for NET mRNA quantification and NET and EEIF for GAPDH mRNA quantification. The siRNA GAPDH was used as a positive control to evaluate the transfection and gene downregulation efficiency. **E** mIBG internalization in IGR-NB8 cells incubated with BGT226 in a dose-dependent manner for 48 h. **F** mIBG internalization following NET and/or DAT downregulation and BGT226 treatment. Statistical analysis was performed through the unpaired t-test using either DMSO (*) or siRNA ctrl (#) as control and comparing with each of the conditions tested. ***p < 0.001; ^##^p < 0.01. **G** mIBG internalization in IGR-NB8 cells incubated with different PI3K, Akt and mTOR inhibitors for 48 h. Statistical analysis was performed through the unpaired t-test between the control and condition tested. **p < 0.01; ***p < 0.001. All experiments were repeated at least three times in duplicates, normalized by a BCA test and expressed as fold of increase or decrease in mIBG internalization compared with non-treated cells

To understand the respective roles of NET and DAT in mIBG internalization following treatment with CUDC-907, we used DMI and GBR12935 specific inhibitors. The addition of DMI 1 μM to CUDC-907 nearly abolished mIBG internalization when compared with control cells (no inhibitors) (fold change = 0.05 ± 0.01, p < 0.0001) (Fig. 4A), while the inhibition of GBR12935 at 1 µM was around 70% (fold change = 0.31 ± 0.14, p < 0.0001). When both inhibitors were added at the same time at high concentrations (0.5 μM and 1 μM, respectively), we observed a similar inhibition profile as when the cells were treated with DMI alone. As already observed in previous experiments, the addition of CUDC-907 0.1 μM increased mIBG internalization threefold (fold change = 3.00 ± 0.76, p < 0.0001) when compared to control. DMI reduced mIBG internalization by nearly 60% at the lowest concentration used while at an increased concentration (up to 1 μM) it nearly abolished mIBG internalization. When GBR12935 0.5 µM was added to the cells in the presence of CUDC-907, it inhibited nearly 50% of mIBG internalization, and the combination of DMI and GBR12935 once again was similar to the profile of inhibition seen with DMI alone. These results suggest that mIBG internalization is mainly performed by NET, even though DAT may also be involved (to a lesser extent) in the internalization process.

To confirm that NET is the main transporter for mIBG internalization, we used a siRNA technology to downregulate NET and/or DAT expression (Fig. 4B). The downregulation of NET led to a decrease of mIBG internalization compared with control cells (siRNA non-targeting sequence) (fold change = 0.58 ± 0.18; p < 0.05), while the downregulation of DAT showed no effect following treatment with CUDC-907 when compared with control cells (fold change = 0.96 ± 0.19, p = 0.79). Finally, when both NET and DAT were downregulated, the profile was similar to the one where only NET expression was inhibited, even though this difference was statistically non significant (p = 0.0964). Quantification of NET mRNA expression by qPCR demonstrated the correlation between a decrease of mIBG uptake and downregulation of NET by siRNA in transfected cells by 2.2 fold (p < 0.0001) compared with cells transfected with siRNA non-targeting sequence (Fig. 4C). DAT mRNA expression was too close or under the limit of quantification to give reliable data (> 33 PCR cycles for control and treated cells).

A decrease of NET expression (1.31 fold, p = 0.001) was also observed in DAT-siRNA transfected cells, probably due to sequence homologies between the two genes. Downregulation of both NET and DAT led to similar results as for NET alone (Fig. 4C) as it is observed for mIBG uptake. The quantification of GAPDH mRNA in siRNA-GAPDH transfected cells was used as a control to evaluate the transfection efficiency (Fig. 4D).

### Assessment of the impact of PI3K/mTOR inhibition on mIBG internalization and the effects on NET and DAT expression

Since CUDC-907 is a dual inhibitor of HDAC and phosphoinositide 3-kinase (PI3K), we explored the role of each transducing pathway separately to assess which one prevailed in the internalization process. BGT226, a dual PI3k/mTOR inhibitor was tested on IGR-NB8 cells and was shown to increase mIBG internalization in a dose-dependent manner (Fig. 4E). At 50 nM, BGT226 increased mIBG internalization by 2.5-fold when compared with non-treated cells (p < 0.01).

We observed that treatment with CUDC-907 and BGT226 exhibited the same potency for mIBG uptake, (3.06 vs 2.47 fold higher than control p = 0.26) suggesting that CUDC-907 effects involve PI3k inhibition (Fig. 4F) but not exclusively since sodium-4-P which is an HDACi with no demonstrated PI3k inhibitory activity showed similar activity to CUDC-907 and BGT226. To further evaluate the effect of BGT226 on NET and DAT, we performed a siRNA downregulation of these transporters separately and simultaneously (Fig. 4G), as previously performed for CUDC-907. The downregulation of NET led to a decrease of 1.85 fold (p < 0.01) in mIBG internalization after treatment with BGT226. Conversely, DAT silencing did not have a significant effect on mIBG internalization. Lastly, silencing of both NET and DAT resulted in the same profile as the NET downregulation alone (twofold, p < 0.01). These results were similar as the one obtained with CUDC-907 incubation.

### Evaluation of the effects of inhibition of different components of the PI3K/AKT/mTOR pathway: effects on mIBG internalization

As PI3K is a component of the AKT/mTOR signaling pathway [28], we further investigated the inhibition of different components of this axis and the effects on mIBG internalization. VS-5584, a PI3K/mTOR inhibitor and rapamycin, a mTOR inhibitor, significantly increased mIBG internalization when compared to non-treated cells (1.51 fold, p < 0.001 and 1.34 fold, p < 0.001, respectively) (Fig. 4H). On the other hand, MK2206, an Akt inhibitor, significantly decreased mIBG internalization when compared to control cells (fold change = 1.38, p < 0.01) (Fig. 4H). None of these inhibitors had toxic effects at the conditions tested (Additional file 1: Fig. S1E).

### Assessment of the impacts of CUDC-907 and BGT226 treatment on NET and DAT protein expression

**Fig. 5:**
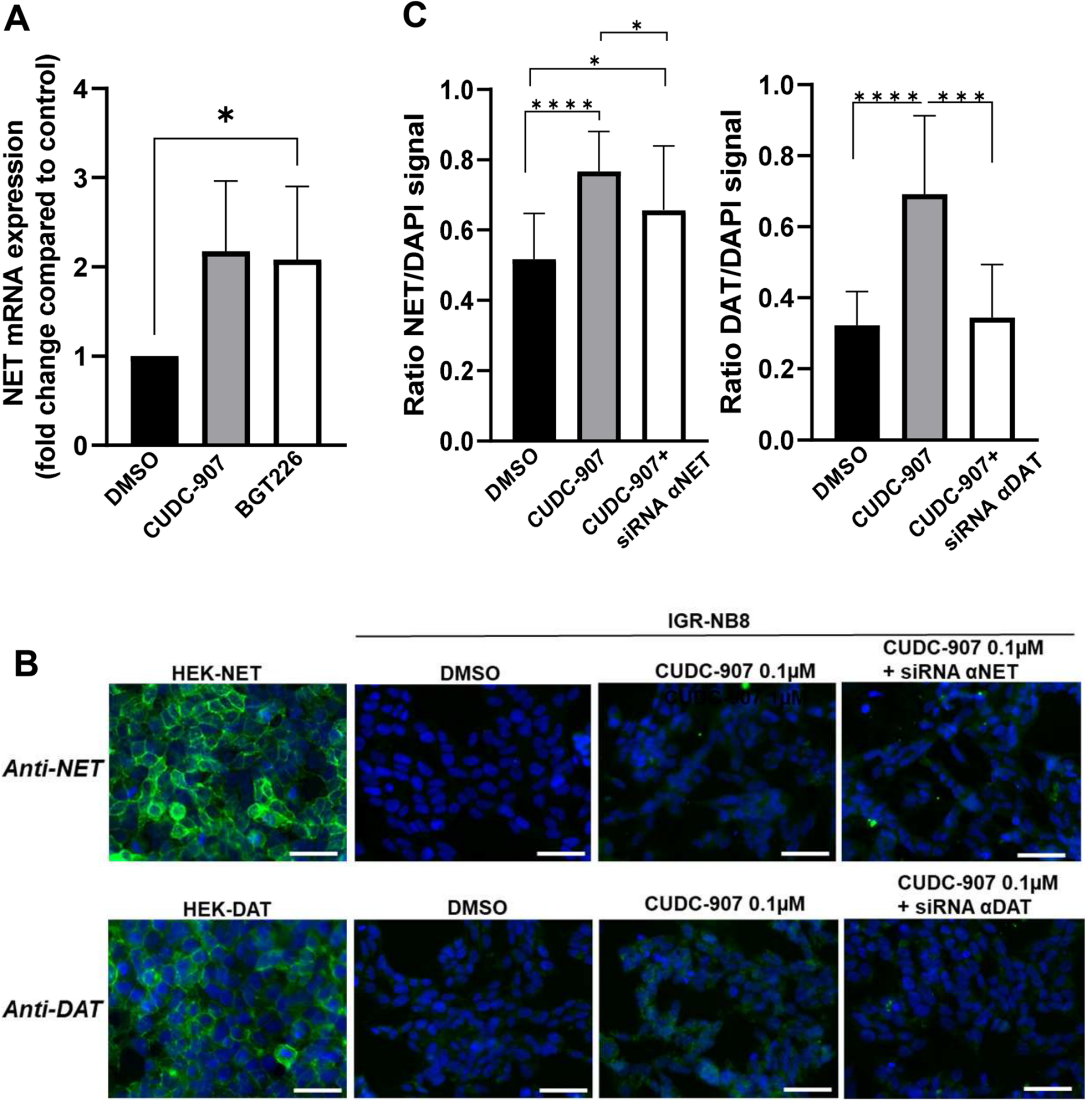
Effects of CUDC-907 on IGR-NB8 cells: NET and DAT mRNA expression and protein levels. **A** NET mRNA expression levels in IGR-NB8 cells incubated with CUDC-907 0.1 µM and BGT226 0.05 µM. The qPCR was performed on duplicate from three independent experiments. Gene expression was detected between 14.9 and 22.6 Ct. **B** Representative immunofluorescence images of NET and DAT protein expression in IGR-NB8 cells incubated with CUDC-907 0.1 µM and CUDC-907 0.1 µM along with siRNA, left panels: positive controls: HEK cells transfected with NET- and DAT-encoding plasmids, scale bars correspond to 50 µM. **C** Quantification of immunofluorescence signal performed in triplicate (three individual fields) for each conditions from at least three independent experiments with the ImageJ software. The ratio corresponds to the NET and DAT signal divided by the signal of the nucleus stain (DAPI) Statistical analysis was performed through the unpaired t-test between the control (DMSO) and CUDC-907 treated cells. *p < 0.05; **p < 0.01

We next investigated the effects of the drugs on NET and DAT expression by RT-qPCR quantification. NET mRNA expression was shown to be significantly increased in CUDC-907-treated cells compared with control cells (fold change = 2.17 ± 0.32, p < 0.05) as well as in BGT226 treated cells (fold change = 2.0 ± 0.33, p < 0.05) (Fig. 5A). DAT mRNA expression was too close or under the limit of quantification to give reliable data (> 33 PCR cycles for control and treated cells). Detection of NET and DAT protein by immunoblot was not possible, probably due to too low expression level, therefore, we performed immunofluorescence detection and observed an increase of NET and DAT protein fluorescence in treated cells (fold change = 1.57 ± 0.29, p < 0.001 and 2.0 ± 0.34, p < 0.001 respectively) (Fig. 5B and C). To demonstrate signal specificity, NET and DAT mRNA was silenced by siRNA and the results showed a significant decrease in NET and DAT protein expression (fold change = 1.25 ± 0.16 p = 0.036 and 1.79 ± 0.3 p < 0.001 respectively (Fig. 5B and C)

### Evaluation of the effects of CUDC-907 treatment on ^123^I-mIBG uptake in vivo

**Fig. 6:**
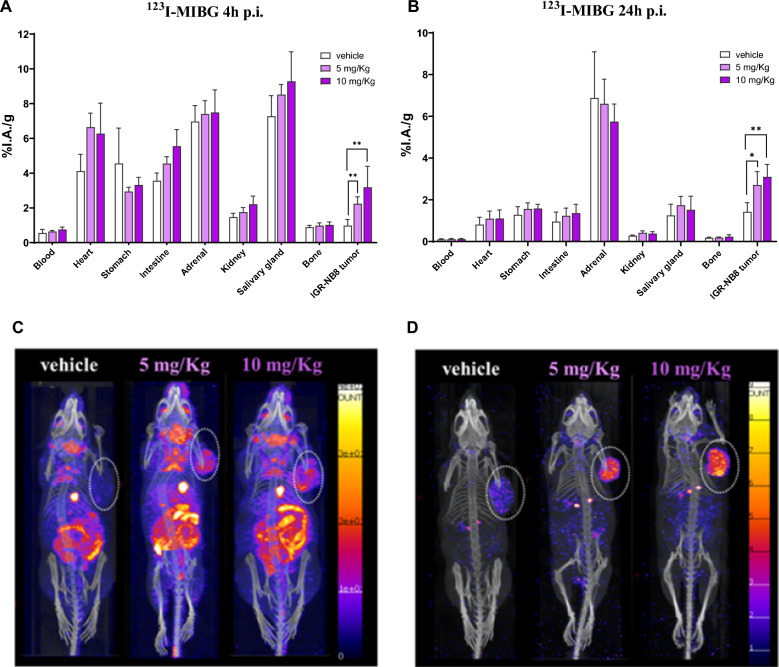
In vivo uptake of ^123^I-mIBG. **A** and **B** Quantitative ex vivo biodistribution data of ^123^I-mIBG (2–4 MBq) in selected organs at 4 h and 24 h p.i. Statistically significant differences were found between the groups concerning accumulation of the radioligand in tumors; more specifically 2.2 and 3.2-fold higher uptake at 4 h p.i. and 1.9 and 2.2-fold higher uptake at 24 h p.i. in the treated groups (5 and 10 mg/kg, respectively) compared to vehicle. **C** and **D** Representative SPECT/CT images of the three treated groups: vehicle (10% DMSO in corn oil), 5 mg/kg CUDC-907 and 10 mg/kg CUDC-907, at 4 h (**C**) and 24 h (**D**) after administration of 15–18 MBq ^123^I-mIBG. The mice were scanned under anesthesia for 90 min (**C**) and the same mice were scanned 24 h later for 3 h, after euthanasia (**D**)

IGR-NB8 xenografted mice were randomly divided into three groups (A: vehicle; B: 5 mg/kg; C: 10 mg/kg) (Additional file 1: Fig. S2). CUDC-907 given orally to the mice for 5 days, was well tolerated with no side effects. After 2 days’ drug-free, biodistribution studies of ^123^I-mIBG were performed at 4 h and 24 h p.i. (Fig. 6A and B). At 4 h p.i., the treated groups showed no significant differences in the accumulation of ^123^I-mIBG in most of the organs and tumors. The administration of the two different doses of CUDC-907 in groups B and C did not significantly impact the tumor uptake of ^123^I-mIBG (2.2 ± 0.4 %IA/g vs. 3.2 ± 1.2%IA/g for group B and C, respectively, p = 0.1). The tumor uptake of both groups was significantly higher when compared to the uptake in the vehicle group (2.2 ± 0.4 %IA/g vs. 1.0 ± 0.3 %IA/g, for group B and A, respectively, p < 0.05) (Fig. 6A). At 24 h p.i., a remarkable background clearance and washout from all organs was observed, except for the adrenal glands (Fig. 6B). Interestingly, no washout from the tumors was observed (tumor uptake was similar between 4 and 24 h p.i.). The tumor uptake remained significantly higher in the treated groups compared to the untreated group (2.7 ± 0.6 %IA/g vs 1.4 ± 0.4 %IA/g, for group B and A, respectively, p < 0.05). These data are detailed in Additional file 1: Table S5.

### Assessment of SPECT/CT imaging of ^123^I-mIBG in treated and untreated mice

Representative SPECT/CT images from each group (A, B and C) were acquired 4 and 24 h (Fig. 6C and D) after the injection of ^123^I-mIBG. The same mouse per group was imaged at both time points, demonstrating the significant improvement in the tumor to background contrast, and consequently clear tumor visualization and delineation, with ^123^I-mIBG over time. At 4 h p.i. ^123^I-mIBG accumulation in the gallbladder and in the intestine was observed, independently of the CUDC-907 treatment regimen (5 mg/kg or 10 mg/kg) (Fig. 6C). At 24 h p.i. there was a complete background clearance, except for the adrenal glands and the tumors, confirming the quantitative biodistribution data (Fig. 6D and Additional file 1: Table S5) and leading to an optimal tumor to background ratio.

## Discussion

mIBG is considered to be mainly internalized by NET [11, 29, 30], However our study on PHEO tissues suggested that other monoamine transporters might be involved in the uptake process, since a discrepancy between NET and DAT protein expression and a positive-^123^I-mIBG signal was observed. Nevertheless, after analysis of other monoamine transporters that could potentially internalize mIBG on the basis of their affinity for catecholamine, we demonstrated that either their affinity for mIBG was too low (PMAT, OCT1–3), or their expression on tumor cells was insufficient (DAT) despite a capacity to internalize mIBG comparable to NET. We also demonstrated for the first time the high affinity of DAT for mIBG which contrasts with a previous study by Glowniak et al. [25]. In that study mIBG was internalized by neither DAT nor serotonin transporter (SERT) in cell lines, however this study was performed on transporters of bovine and rat origin, respectively [25]. SERT was not considered in our study due to its low affinity for norepinephrine and because it is not involved in tumoral mIBG uptake, as recently demonstrated [31]. We thus demonstrated that in some patients, the lack of correlation between NET concentration and mIBG uptake could be due to other reasons that the activity of other monoamine transporters, such as non-compliance with the instructions before the scan (the use of certain drugs e.g. tricyclic antidepressant may interfere with uptake), a residual amount of these drugs present in the plasma of patients who followed the instructions or the fact that a very low expression of NET is sufficient to internalize the mIBG. Others reports on neuroblastoma and pheochromocytoma patients have also demonstrated an overlap between NET protein expression and mIBG avid and mIBG nonavid tumors [11–13].

In our cell system, inhibition of NET and DAT expression (by siRNA) or activity (with specific inhibitors) demonstrated that DAT plays only a minor role in the cellular uptake of mIBG, which is certainly explained by the fact that IGR-NB8 cells express only trace amounts of DAT. However, considering the heterogeneity of tumors, some may express high amounts of DAT and therefore be able to efficiently internalize mIBG as observed for DAT-transfected HEK cells.

We then screened a panel of HDACi to find a compound able to increase the expression of NET and DAT and consequently improve the uptake of mIBG in cell culture. More et al*.* described that vorinostat was able to increase NET mRNA and protein levels in Kelly and SH-SY-5Y NB cells, as well as demonstrated a 4- and 2.5-fold increase in mIBG internalization, respectively [18]. The in vivo proof of concept was successfully performed using a xenografted mice model [18]. In our study, we identified CUDC-907 (fimepinostat), as the best enhancer of mIBG uptake in another NB cell line, namely IGR-NB8. Our findings were confirmed in two other NB human cell lines and in a cell line from another neuroendocrine tumor (PC12 from rat pheochromocytoma). This shows that distinct and multiple types of neuroendocrine tumors may respond to treatment with CUDC-907 as a radiosensitizer before mIBG administration.

CUDC-907 is a dual inhibitor of PI3K (class I α, β and δ) and HDAC (HDAC 1, 2, 3, 6, 10 and 11). It is known as an anticancer agent, inducing DNA damage, cell cycle arrest and apoptosis in breast and prostate cancer cells, and it works synergistically with other anticancer agents for the treatment of acute myeloid leukemia [32–34]. Using specific inhibitors of PI3K (VS5584) of mTOR (rapamycin) and a dual PI3K/mTOR inhibitor (BGT226), we demonstrated that mIBG uptake was also increased by inhibition of this pathway. Therefore, our data suggest that both pathways (PI3K/AKT/mTOR and HDAC) are involved in mIBG uptake, on the other hand, Akt inhibition by MK2206 decreases mIBG internalization. This could be explained by the complexity of the regulatory mechanism exerted on Akt by proteins or complexes acting downstream of Akt itself [35–37] and in pathways parallel to the PI3K/AKT/mTOR axis. Inhibition of Pi3K and mTOR could induce increased internalization (presumably via activation of NET synthesis) by mechanisms distinct from and therefore independent of Akt. In this scenario, inhibition of Akt would activate a regulatory countermechanism on NET, albeit with less efficacy considering the weak inhibition of internalization. MIBG after MK2206 treatment. Another report demonstrated that NET regulation by insulin is Akt-dependent since Akt inhibition significantly increased NET surface expression in a time-dependent manner [35]. The same study found that inhibition of mTORC2 (the kinase responsible for Akt phosphorylation and subsequent activation) resulted in increased NET expression, which was also reported in another more recent study [36]. The mechanistic link between the Akt pathway, HDACi-coupled epigenetic modifications, and NET expression is still unclear. However, it has been hypothesized that the increase in NET levels observed following exposure to an HDACi (vorinostat) may occur through the disruption of HDAC-protein phosphatase 1 interactions, resulting in the dephosphorylation of Akt at serine 473 [37]. Monoamine transporters are phosphorylated by kinases, and it has been shown that changes in phosphorylation status correlate with transporter activity [38–40]. It is therefore possible that modulation of PI3K/AKT/mTOR may affect not only the synthesis of monoamine transporters at the mRNA or protein level, but also their activity (in our case mIBG uptake) modulated by phosphorylation. As observed in our experiments on cultured cells, mIBG has the advantage of remaining in neuroblastoma cells after its specific internalization by NET, which is not the case for most cells in the various organs considered in mice after 24 h following mIBG injection. Chromaffin cells, mainly concentrated in the adrenal glands, allow NET to internalize high concentrations of mIBG, which should clearly be taken into account when looking for neuroblastomas. It would be very interesting to induce increased mIBG uptake via a similar strategy (HDACI and PI3K inhibitors) on pheochromocytoma cells, and thus improve detection of this type of cancer, particularly in the case of malignant tumors.

Recently, CUDC-907 has been demonstrated to inhibit both HDAC1 and HDAC2 as well as PI3K, Akt and mTOR expression and this resulted in an inhibition of NB tumor growth in a 3D spheroid tumor model [41]. In another report, CUDC-907 was shown to restrain the growth of gallbladder carcinoma organoids via restrained AkT and HDAC activities [42]. Different treatment regimens of CUDC-907 in xenografted models have also been extensively studied, demonstrating the broad antitumor activities of CUDC-907 [43]. Our experimental protocol was designed to study this inhibitor as a pharmacological inducer of the transporters involved in mIBG uptake, and not as a therapeutic drug. Therefore, administered doses and treatment regimens were weakened, compared to the ones reported in the literature [44]. The low dose of 5 mg/kg of CUDC-907, under the reported treatment regimen, showed to be already very efficient in ^123^I-mIBG uptake; induced an increase in the tumor uptake of 2.2 and 1.9 times higher (at 4 h and 24 h p.i., respectively), compared to control group.

Regarding the therapeutic aspects of NB, vorinostat as a radiosensitizer was shown in a recent phase II clinical trial to increase the response rate of patients receiving ^131^I-mIBG [45]. The initial proof of concept of this study was performed using an approach similar to the one presented in this report. However, in our study, vorinostat did not show significant effects on mIBG internalization in IGR-NB8 cells, which is potentially related to the different cell lines used in the two studies. Nevertheless, given the remarkable results obtained in our mouse model, a clinical trial may be considered following a similar protocol involving HDAC and PI3K as a new target to improve theranostics of these tumors.

## Conclusions

Radiolabeled mIBG is considered the standard of care method for staging and monitoring treatment response in patients with NB, based on a well-defined scoring system. In addition, mIBG has been used as a systemic tumor targeting treatment for relapsed/refractory disease or as palliative care, and more recently as promising front-line treatment in high-risk patients, alone or in combination with chemotherapeutic agents. In this study we demonstrate that enhancing internalization of mIBG into tumor cells not only by HDACi but also by PI3K/AKT/mTOR inhibitors that represent a novel target and could therefore improve diagnostic precision in a larger number of patients improving efficacy of mIBG as a major therapeutic modality.

### Supplementary Information

**Additional file 1: Table S1.** Patients and tumors characteristics: Sp: sporadic, Mutation investigated: RET: Rearranged after transfection (Multiple Endocrine Neoplasia type 2 syndrome), SDH: succinate dehydrogenase subunits A, B, C and D, SDHAF2: succinate dehydrogenase complex assembly factor 2, VHL: von Hippel-Lindau, MAX: MYC-associated factor X. **Table S2.** List of the different inhibitors used and respective references. **Table S3.** Primers used for RT-qPCR. **Table S4.** Chromatographic gradient used for the separation of mIBG by LC*–*MS/MS. **Table S5.** Biodistribution data and SPECT/CT images of ^123^I-mIBG in IGR-NB8 xenografts at 4 h and 24 h p.i. **Figure S1.** Effect of the treatment with different inhibitors on cell viability in IGR-NB8, SK-N-Be2C, LAN-1 and PC-12 cells. **Figure S2.** Experimental design of the* in vivo *studies.

**Additional file 2.** Additional data.

## Data Availability

All data generated or analyzed during this study are included in this published article (and its additional information files).

## Abbreviations

NB: Neuroblastoma
PHEO/PGL: Pheochromocytoma/paraganglioma
mIBG: Meta-iodobenzylguanidine
HEK: Human embryonic kidney
HDACi: Histone deacetylase inhibitors
PI3K/AKT/mTOR: Phosphatidylinositol-3-kinase/AKT/mammalian target of rapamycin
DAT: Dopamine transporter
NET: Norepinephrine transporter
PMAT: Plasma membrane monoamine transporters
OCT1–3: Organic cation transporters 1–3
DMI: Desipramine
BSA: Bovine serum albumin
DMEM: Dulbecco’s modified eagle medium
SDS: Sodium dodecyl sulfate
PCR: Polymerase chain reaction
RT-qPCR: Real time quantitative PCR
DMSO: Dimethyl sulfoxide
GAPDH: Glyceraldehyde 3-phosphate dehydrogenase
EEIF1A1: Eukaryotic translation elongation factor 1 alpha 1
BCA: Bicinchoninic acid
LC–MS/MS: Liquid chromatography tandem mass spectrometry
SPECT: Single-photon emission computerized tomography
Km: Michaelis–Menten constant
Vmax: Maximal velocity
IA/g: Radioactivity per gram of tissue
SERT: Serotonin transporter
