# Supplementary material for: CUDC-907, a dual PI3K/histone deacetylase inhibitor, increases meta-iodobenzylguanidine uptake (123/131I-mIBG) in vitro and in vivo: a promising candidate for advancing theranostics in neuroendocrine tumors
Source: J Transl Med. 2023 Sep 7;21:604. doi: 10.1186/s12967-023-04466-z (PMC10485979; doi:10.1186/s12967-023-04466-z)
Supplement: Supplementary file 1 — Additional file 1: Table S1. Patients and tumors characteristics: Sp: sporadic, Mutation investigated: RET: Rearranged after transfection (Multiple Endocrine Neoplasia type 2 syndrome), SDH: succinate dehydrogenase subunits A, B, C and D, SDHAF2: succinate dehydrogenase complex assembly factor 2, VHL: von Hippel-Lindau, MAX: MYC-associated factor X. Table S2. List of the different inhibitors used and respective references. Table S3. Primers used for RT-qPCR. Table S4. Chromatographic gradient used for the separation of mIBG by LC–MS/MS. Table S5. Biodistribution data and SPECT/CT images of 123I-mIBG in IGR-NB8 xenografts at 4 h and 24 h p.i. Figure S1. Effect of the treatment with different inhibitors on cell viability in IGR-NB8, SK-N-Be2C, LAN-1 and PC-12 cells. Figure S2. Experimental design of the in vivo studies. [file 12967_2023_4466_MOESM1_ESM.docx]

**Additional Tables and Figures**

**Table S1.** Patients and tumors characteristics: Sp: sporadic, Mutation investigated: RET: Rearranged after transfection (Multiple Endocrine Neoplasia type 2 syndrome), SDH: succinate dehydrogenase subunits A, B, C and D, SDHAF2: succinate dehydrogenase complex assembly factor 2, VHL: von Hippel-Lindau, MAX: MYC-associated factor X.

**Table S2.** List of the different inhibitors used and respective references.

| Inhibitors | Reference |
| --- | --- |
| ACY-1215 | **MedChem Express (HY-16026)** |
| CUDC-101 | **Selleckchem (S1194)** |
| CUDC-907 | **MedChem Express (HY-13522)** |
| Entinostat | **MedChem Express (HY-12163)** |
| LMK-235 | **MedChem Express (HY-18998 )** |
| Mocetinostat | **MedChem Express (HY-12164)** |
| PCI-24781 | **MedChem Express (HY-10990)** |
| Quisinostat | **Selleckchem (S1096)** |
| Romidepsin | **MedChem Express (HY-15149)** |
| Sodium-4-Phenylbutyrate | **Sigma-Aldrich (567616)** |
| Tacedinaline | **Selleckchem (S2818)** |
| Trichostatin A | **Reagents Direct (62-K44)** |
| Tubacin | **MedChem Express ( HY-13428)** |
| Tucidinostat | **MedChem Express ( HY-109015)** |
| Valproic acid | **Sigma-Aldrich (676380)** |
| Vorinostat | **Sigma-Aldrich (SML0061)** |
| BGT226 | **MedChem Express ( HY-13334)** |
| VS-5584 | **MedChem Express ( HY-16585)** |
| MK2206 | **MedChem Express ( HY-108232)** |
| Desipramine | **Sigma-Aldrich (D3900)** |
| GBR12935 | **Sigma-Aldrich (G9659)** |
| Rapamycin | **Sigma-Aldrich (37094)** |

**Table S3.** Primers used for RT-qPCR.

| Primers | Sequence |
| --- | --- |
| hNET Forward | 5’ TGA TGG GGT TCA GGC CGG GT 3’ |
| hNET Reverse | 5’ TTG GGC CAG GGC GGG AAG AT 3’ |
| hDAT Forward | 5’ CCA TAG ACG GCA TCA GAG CA 3’ |
| hDAT Reverse | 5’ CCG CGT CAA TCC AAA CAG A 3’ |
| hOCT1 Forward | 5’ TTT GGC CGT AAG CTG TGT CT 3’ |
| hOCT1 Reverse | 5’ ATG TAG TTG GGC GAG AAG GC 3’ |
| hOCT2 Forward | 5’ TGC AGC TGG AGT TCT CAT GG 3’ |
| hOCT2 Reverse | 5’ TGC TTT GCT GAC CAG TCC TT 3’ |
| hOCT3 Forward | 5’ CCC CAA ATG AGG AAA TGC ACA 3’ |
| hOCT3 Reverse | 5’ CAC CAC TGC GCT TGT GAA 3’ |
| hPMAT Forward | 5’ CGC CAT CTA CTT TGC GAT GC 3’ |
| hPMAT Reverse | 5’ ACG TCC GTG ATG AAG CTG TT 3’ |
| hGAPDH Forward | 5’ CAT CCA TGA CAA CTT TGG TAT CGT 3’ |
| hGAPDH Reverse | 5’ CCA TCA CGC CAC AGT TTC C 3’ |
| hEEIF1A1 Forward | 5’ CTG AAC CAT CCA GGC CAA AT 3’ |
| hEEIF1A1 Reverse | 5’ GCC GTG TGG CAA TCC AAT 3’ |

**Table S4.** Chromatographic gradient used for the separation of mIBG by LC-MS/MS.

| Time (min) | Flow (mL/min) | % solution A | % solution B |
| --- | --- | --- | --- |
| Initial | 0.350 | 5.0 | 95.0 |
| 4.00 | 0.350 | 30.0 | 70.0 |
| 6.00 | 0.350 | 30.0 | 70.0 |
| 6.10 | 0.350 | 5.0 | 95.0 |
| 9.00 | 0.350 | 5.0 | 95.0 |

**Table S5.** Biodistribution data and SPECT/CT images of ^123^I-mIBG in IGR-NB8 xenografts at 4 hours and 24 hours p.i.

| ^123^I-mIBG (IGR-NB8 xenografts) | | | | | | |
| --- | --- | --- | --- | --- | --- | --- |
|  | **4h p.i.** | | | **24h p.i.** | | |
| Organs | **Vehicle* (n=3)** | **CUDC-907 5 mg/Kg (n=5)** | **CUDC-907 10 mg/Kg** **(n=8)** | **Vehicle* (n=4)** | **CUDC-907 5 mg/Kg (n=4)** | **CUDC-907 10 mg/Kg** **(n=5)** |
| Blood | 0.5 ± 0.2 | 0.6 ± 0.1 | 0.8 ± 0.1 | 0.1 ± 0.03 | 0.1 ± 0.04 | 0.1 ± 0.03 |
| Heart | 4.1 ± 1.0 | 6.6 ± 0.8 | 6.3 ± 1.8 | 0.8 ± 0.3 | 1.1 ± 0.4 | 1.1 ± 0.4 |
| Lung | 3.2 ± 0.8 | 3.1 ± 0.4 | 3.4 ± 0.5 | 0.4 ± 0.1 | 0.4 ± 0.1 | 0.4 ± 0.1 |
| Liver | 3.4 ± 0.5 | 3.7 ± 0.5 | 4.0 ± 0.4 | 0.4 ± 0.1 | 0.6 ± 0.1 | 0.5 ± 0.2 |
| Pancreas | 2.1 ± 0.4 | 2.4 ± 0.2 | 2.8 ± 0.5 | 0.4 ± 0.1 | 0.4 ± 0.04 | 0.4 ± 0.1 |
| Spleen | 2.6 ± 0.9 | 2.1 ± 0.6 | 2.8 ± 0.4 | 0.7 ± 0.3 | 0.6 ± 0.2 | 0.7 ± 0.2 |
| Stomach | 4.6 ± 2.0 | 2.9 ± 0.2 | 3.3 ± 0.4 | 1.3 ± 0.4 | 1.6 ± 0.3 | 1.6 ± 0.2 |
| Intestine | 3.6 ± 0.4 | 4.5 ± 0.4 | 5.5 ± 1.0 | 0.9± 0.5 | 1.2 ± 0.4 | 1.4 ± 0.4 |
| Adrenal | 7.0 ± 0.9 | 7.4 ± 0.8 | 7.5 ± 1.3 | 6.9 ± 2.2 | 6.6 ± 1.2 | 5.7 ± 0.8 |
| Kidney | 1.5 ± 0.2 | 1.8 ± 0.3 | 2.2 ± 0.5 | 0.3 ± 0.03 | 0.4 ± 0.1 | 0.4 ± 0.1 |
| Muscle | 1.3 ± 0.3 | 1.1 ± 0.1 | 1.2 ± 0.3 | 0.2 ± 0.03 | 0.2 ± 0.02 | 0.2 ± 0.05 |
| Bone | 0.9 ± 0.1 | 1.0 ± 0.2 | 1.0 ± 0.2 | 0.2 ± 0.03 | 0.2 ± 0.03 | 0.2 ± 0.1 |
| IGR-NB8 tumor | **1.0 ± 0.3 (p<0.05)** | **2.2 ± 0.4** | **3.2 ± 1.2 (p=0.1)** | **1.4 ± 0.4**  **(p<0.05)** | **2.7 ± 0.6** | **3.1 ± 0.6 (p=0.4)** |
| Salivary gland | 7.3 ± 1.2 | 8.5 ± 0.6 | 9.3± 1.7 | 1.2 ± 0.5 | 1.7 ± 0.4 | 1.5 ± 0.6 |

**
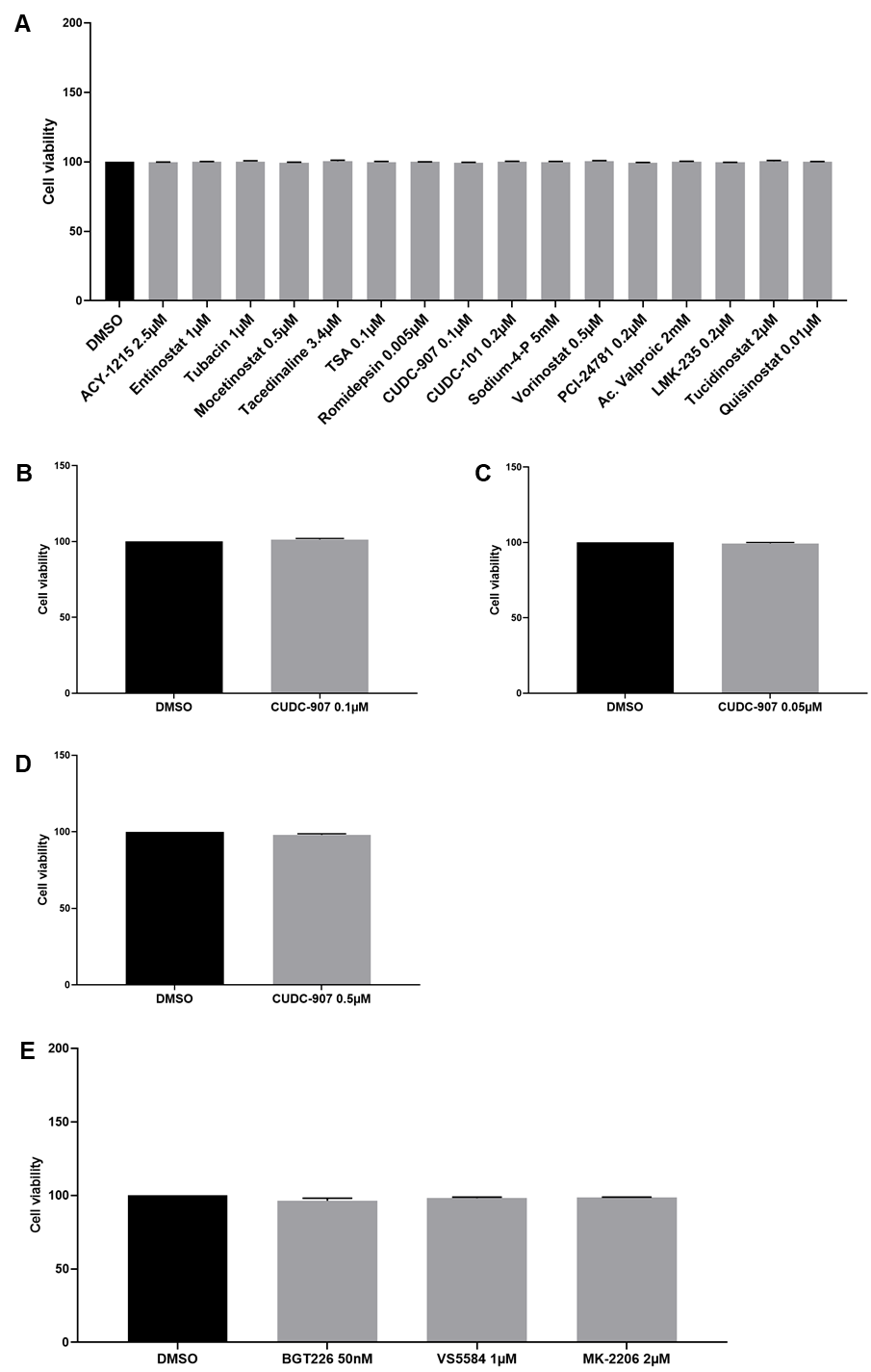
**

**Figure S1. Effect of the treatment with different inhibitors on cell viability in IGR-NB8, SK-N-Be2C, LAN-1 and PC-12 cells.**

**A)** IGR-NB8 cells were incubated with different HDACi for 48 hours. **B)** SK-N-Be2C cells were incubated with CUDC-907 0.1 µM for 48 hours. **C)** LAN-1 cells were incubated with CUDC-907 0.05 µM for 48 hours. **D)** PC-12 cells were incubated with CUDC-907 0.5 µM for 48 hours. **E)** IGR-NB8 cells were incubated with PI3K, Akt and mTOR inhibitors for 48 hours. For all the experiments, after CUDC-907 incubation, a trypan blue assay was performed. All the experiments were performed once in triplicate.


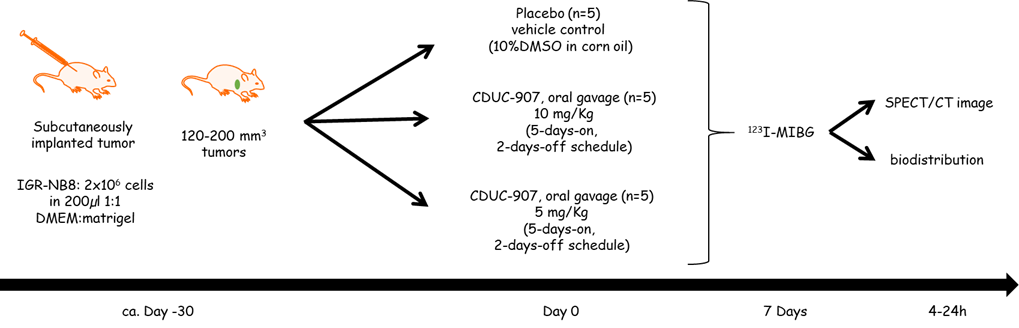


**Figure S2. Experimental design of the in vivo studies.**

IGR-NB8 xenografted mice were randomly divided into three groups (placebo; CUDC-907 10 mg/kg; CUDC-907 5 mg/kg). CUDC-907 was given orally to the mice for 5 days and 2 days drug free.
