# Supplementary material for: CUDC-907, a dual PI3K/histone deacetylase inhibitor, increases meta-iodobenzylguanidine uptake (123/131I-mIBG) in vitro and in vivo: a promising candidate for advancing theranostics in neuroendocrine tumors
Source: J Transl Med. 2023 Sep 7;21:604. doi: 10.1186/s12967-023-04466-z (PMC10485979; doi:10.1186/s12967-023-04466-z)
Supplement: Supplementary file 2 — Additional file 2. Additional data. [file 12967_2023_4466_MOESM2_ESM.docx]

**Additional data**

**Additional Material and Methods**

RNA extraction and RT-qPCR

The primers described in Supplementary Table 3 were designed using the “Primer Blast” tool from the NCBI website. Reactions were performed in a QuantStudio 6 Real-Time PCR System, and amplification was carried out in a 384-well reaction plate as follows: 95°C for 10 minutes, followed by 40 cycles of 95°C for 15 seconds and 60°C for 1 minute. The limit of quantification values was set at 33 cycles. Each sample was analyzed in duplicate, and a negative control was prepared by using the same amount of total RNA without adding the enzyme transcriptase reverse.

**Proteomic**

*Sample preparation*

Protein samples were separated by SDS-PAGE 12% and stained by Coomassie. Gel lanes were quantified by densitometry and cut between 55-120 kDa. Gel bands were digested with sequencing-grade trypsin. Extracted tryptic peptides were dried and resuspended in 0.05% trifluoroacetic acid, 2% (v/v) acetonitrile.

*Mass spectrometry analyses*

Tryptic peptide mixtures were injected on an Ultimate RSLC 3000 nanoHPLC system interfaced via a nanospray Flex source to a high resolution QExactive Plus mass spectrometer (Thermo Scientific). Amounts injected were normalized based on gel densitometry results. Peptides were loaded onto a trapping microcolumn Acclaim PepMap100 C18 (20 mm x 100 μm ID, 5 μm, Thermo Scientific) before separation on a C18 custom packed column (75 μm ID × 50 cm, 1.8 μm particles, Reprosil Pur, Dr. Maisch), using a gradient from 4 to 76 % acetonitrile in 0.1 % formic acid for peptide separation (total time: 140 min). Full MS survey scans were performed at 70,000 resolution. In data-dependent acquisition controlled by Xcalibur software (Thermo Scientific), the 10 most intense multiply charged precursor ions detected in the full MS survey scan were selected for higher energy collision-induced dissociation (HCD, normalized collision energy NCE=27 %) and analysis in the orbitrap at 17’500 resolution. The window for precursor isolation was of 1.5 m/z units around the precursor and selected fragments were excluded for 60s from further analysis.

*Data analysis*

Tandem MS data were processed by the MaxQuant software(46) (version 1.6.3.4) incorporating the Andromeda search engine(47). The UniProt *Homo sapiens* reference proteome database of October 29^th^, 2017 was used (71’803 sequences), supplemented with sequences of common contaminants. Trypsin (cleavage at K,R) was used as the enzyme definition, allowing 2 missed cleavages. Carbamidomethylation of cysteine was specified as a fixed modification. N-terminal acetylation of protein and oxidation of methionine were specified as variable modifications. All identifications were filtered at 1% FDR at both the peptide and protein levels with default MaxQuant parameters. MaxQuant data were further processed with Perseus software(48). LFQ values (49) were used for quantitation after log2 transformation.

**mIBG extraction and LC-MS/MS**

Intracellular mIBG was extracted by solid-phase extraction performed on Waters Oasis WCX μElution 96-well plates (Waters), preconditioned with 200 µL of methanol and equilibrated with 200 µL of PBS. A total of 30 µL or 40 µL of an internal standard solution at 2 nM (deuterated mIBG), and 30 µL or 40 µL of each sample were loaded and washed three times for HEK cell and IGR-NB8 cell experiments, respectively. A first wash with 200 µL of water, then with 200 µL of methanol and finally with 200 µL of 0.2% formic acid in acetonitrile. Then, the analytes were eluted with a solution containing 2% formic acid in acetonitrile:water (95:5) in 350 μL 96‐well plates or in conical 700 μL 96‐well plates for HEK cell experiments and IGR-NB8 cell experiments, respectively. Separations were performed in HILIC mode on a Waters Acquity UPLC I‐class system (Waters) where 2 μL (HEK cell experiments) or 10 μL (IGR-NB8 cell experiments) of sample were injected on a silica column (Interchim Uptisphere Strategy 100 Å HILIC, 100 mm × 2.1 mm, 2.2 μm) (Alsachim). The mobile phases consisted of 100% acetonitrile (A) and 100 mM ammonium formate (B). The gradient and flow rates are described on Supplementary Table 4. A solution containing 50%, 95% and 5% of acetonitrile was used for the strong, weak and seal washes, respectively. The temperature of the autosampler and the column were 10°C and 25°C, respectively. A Waters Xevo TQ‐S triple quadrupole mass spectrometer equipped with an electrospray interface was coupled to the LC system, and the analyses were performed in a positive ionization mode. The MRM transitions used for quantification were 275.97 (m/z) and 89.93 (m/z) for the precursor ion and product ion, respectively, with cone voltage at 34 V and collision energy at 20 V. The ESI conditions were set as follows: capillary voltage 0.60 kV, desolvation temperature 600°C, source temperature 150°C, desolvation gas flow 900 L/h, cone gas flow 150 L/h, nebulizer gas 7.0 bar, and collision gas flow 0.25 mL/min. At the beginning of each series, a calibration curve was injected, and three quality controls samples were randomly injected. Data was processed using the TargetLynx module.

**SPECT/CT imaging**

SPECT images were reconstructed iteratively and filtered using the HiSPECT software package (version 1.4.1876, SciVis GmbH) and the manufacturer’s algorithm (three subsets, nine iterations, 35% post-filtering, 128 × 128 matrix, and zoom 1.30 x 20 mm transaxial field of view, resulting in a pixel size of 0.3 mm). CT images were reconstructed using CTReco (version r1.146), with a standard filtered back projection algorithm (exact cone beam) and post-filtered (RamLak, 100% frequency cut-off), resulting in a pixel size of 0.2 mm. Co-registered images were visualized in the three orthogonal planes using maximum intensity projection with InVivoScope (version 1.43, Bioscan Inc.).
